# Sperm‐dependent asexual species and their role in ecology and evolution

**DOI:** 10.1002/ece3.10522

**Published:** 2023-09-28

**Authors:** Karel Janko, Peter Mikulíček, Roman Hobza, Ingo Schlupp

**Affiliations:** ^1^ Laboratory of Non‐Mendelian Evolution, Institute of Animal Physiology and Genetics Academy of Sciences of the Czech Republic Liběchov Czech Republic; ^2^ Department of Biology and Ecology, Faculty of Science University of Ostrava Ostrava Czech Republic; ^3^ Department of Zoology, Faculty of Natural Sciences Comenius University in Bratislava Bratislava Slovakia; ^4^ Department of Plant Developmental Genetics, Institute of Biophysics Academy of Sciences of the Czech Republic Brno Czech Republic; ^5^ Department of Biology University of Oklahoma Oklahoma Norman USA; ^6^ Department of Biology International Stock Center for Livebearing Fishes Oklahoma Norman USA

**Keywords:** apparent competition, hybridization, meiosis, population dynamics, speciation

## Abstract

Sexual reproduction is the primary mode of reproduction in eukaryotes, but some organisms have evolved deviations from classical sex and switched to asexuality. These asexual lineages have sometimes been viewed as evolutionary dead ends, but recent research has revealed their importance in many areas of general biology. Our review explores the understudied, yet important mechanisms by which sperm‐dependent asexuals that produce non‐recombined gametes but rely on their fertilization, can have a significant impact on the evolution of coexisting sexual species and ecosystems. These impacts are concentrated around three major fields. Firstly, sperm‐dependent asexuals can potentially impact the gene pool of coexisting sexual species by either restricting their population sizes or by providing bridges for interspecific gene flow whose type and consequences substantially differ from gene flow mechanisms expected under sexual reproduction. Secondly, they may impact on sexuals' diversification rates either directly, by serving as stepping‐stones in speciation, or indirectly, by promoting the formation of pre‐ and postzygotic reproduction barriers among nascent species. Thirdly, they can potentially impact on spatial distribution of species, via direct or indirect (apparent) types of competition and Allee effects. For each such mechanism, we provide empirical examples of how natural sperm‐dependent asexuals impact the evolution of their sexual counterparts. In particular, we highlight that these broad effects may last beyond the tenure of the individual asexual lineages causing them, which challenges the traditional perception that asexual lineages are short‐lived evolutionary dead ends and minor sideshows. Our review also proposes new research directions to incorporate the aforementioned impacts of sperm‐dependent asexuals. These research directions will ultimately enhance our understanding of the evolution of genomes and biological interactions in general.

## Graphical abstract

Asexual species play important roles in the ecology and evolution of ecosystems. Our review highlights the many ways asexuals can influence sexual species.

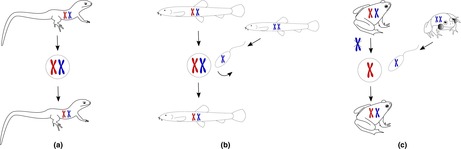

## 1 INTRODUCTION

Broadly speaking, reproduction, the ability to transmit genomes—or heritable information—from one generation to another, is a fundamental property of all living organisms.

**FIGURE 1:**
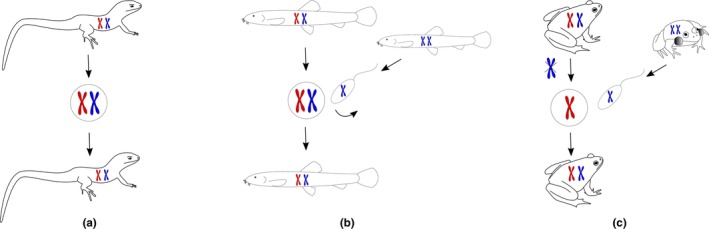
Selected modes of asexual reproduction in animals. Since asexual reproduction might be linked with interspecific hybridization, asexual forms in this scheme are hybrids (red and blue genomes originate from the parental species). (a) A parthenogenetic hybrid female (e.g., in lizards of the genus *Darevskia*) forms unreduced eggs from which a new generation of clonal daughters originate without any contribution of males. (b) Gynogenesis (e.g., in the genus *Cobitis* or *Poecilia*) is a similar mode of reproduction during which unreduced eggs must be activated by sperm of a sexual male to initiate embryogenesis. The sperm, however, do not fertilize the eggs and a new generation of daughters is clonal. (c) A hybridogenetic hybrid female (e.g., in the *Pelophylax esculentus* complex) eliminates a genome of one parental species during gametogenesis and forms clonal eggs which are fertilized by sperm of a sexual male.

**FIGURE 2:**
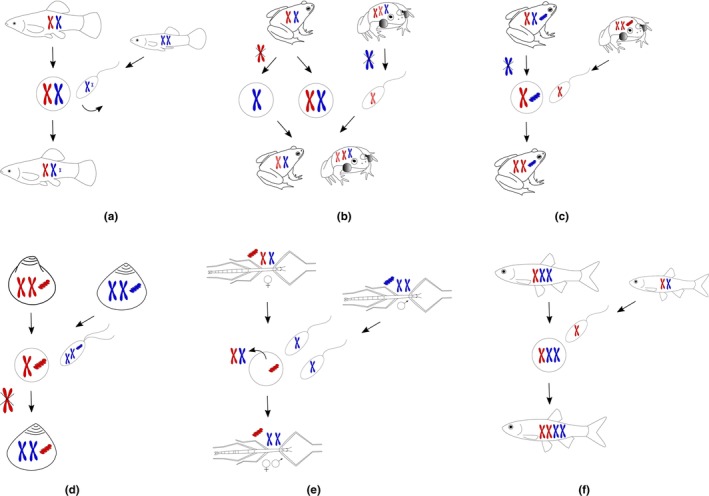
Mechanisms of nuclear (a, b) and mitochondrial (c–e) introgression and polyploid speciation (f) in hybrid asexual complexes (red and blue genomes denote parental species). (a) In gynogenetic systems (e.g., in the fish *Poecilia formosa*), microchromosomes (B chromosomes) might be incorporated into the clonal egg from the paternal sperm. (b) In triploid forms (e.g., in hybridogenetic frogs *P. esculentus*) two different genomes belonging to one parental species (red ones in the scheme) can enter meiosis and recombine after premeiotic elimination of the genome of another parental species (blue genome). This process is called meiotic hybridogenesis. Here mating between a diploid hybrid female producing both diploid and haploid eggs and a triploid male is shown. This mating leads to diploid and triploid progeny of both sexes (in the scheme a male is triploid, and a female is diploid just for simplicity) and enables the perpetuation of a population without the contribution of the parental species. (c) Mating between hybridogenetic hybrids *P. esculentus* and parental species leads to the origin of the parental species progeny with introgressed mtDNA. (d) In androgenetic *Corbicula* clams (see *Androgenesis* in Glossary), the egg is fertilized by an unreduced biflagellate sperm. The entire maternal nuclear genome is then extruded from the oocyte, whereas mitochondria and other organelles from the egg are retained. Thus, the offspring inherit paternal nuclear genome and maternal mtDNA. (e) In the hybrid *Bacillus* stick insect reproducing by androgenesis, the whole nuclear genome is lost from the egg. A fusion of two sperm nuclei occurs because fertilized *Bacillus* eggs contain several spermatozoa (physiological polyspermy). This leads to a sexually reproducing progeny of one parental species with an introgressed mitogenome. (f) Incorporation of sperm into clonal triploid eggs (observed in the *Squalius alburnoides* complex) can lead to sexual tetraploid progeny, which is reproductively isolated from other ploidy forms.

There are, however, a number of variations on this theme. Within prokaryotes, many ways to pass on heritable information, either from parent to offspring or horizontally between individuals are known (Koonin et al., 2001; Yadav et al., 2023). In the world of eukaryotes, such a transmission may be realized via budding, vegetative propagation, or other processes not involving gametes, but gametic reproduction is omnipresent. Generally, the production of gametes involves meiotic sex with recombination, which probably had a single origin very early in eukaryotic evolution (Bernstein & Bernstein, 2010; Mirzaghaderi & Hörandl, 2016). There is, however, considerable variability in the ways how organisms produce their gametes as many transmit parts of their genomes or the whole genomes clonally, commonly being referred to as *asexuals*. While *sex* and *asexuality* (see sexual and asexual reproduction in Glossary) are usually presented as a dichotomy, but in reality, there is a continuum between full meiosis and *mixis* (Glossary) on the one hand, and ameiotic formation of gametes on the other hand. In fact, many sexual species—including our own—pass on parts of their genome, such as mitochondria and sex chromosomes or germ‐line restricted chromosomes, essentially in an asexual mode with no or very limited recombination. The term asexuality is, thus, used very broadly to capture a large diversity of reproductive mechanisms that differ in some aspect from full‐blown sexual reproduction with meiosis and recombination in every generation (Neiman et al., 2014). The strict definition of an “asexual” reproductive mode is, therefore, elusive and necessarily subjective. For instance, Bengtsson (2009) defined asexuals as organisms that have evolved from sexual species by losing regular meiosis and sex and do not alternate sexual and asexual phases throughout their life cycles. In this review, we adopt this definition from Bengtsson (2009), with the modification that such organisms may indeed exhibit “normal” meiosis from the mechanistic point of view (e.g., Dedukh et al., 2020; Kuroda et al., 2018; Marta et al., 2023), but owing to particular inheritance pathways, or premeiotic stages (Figures 1 and 2) their genomes are passed to gametes without efficient recombination.

Even among such organisms that obligately abandoned the canonical sex and Mendelian heredity, there is a wide spectrum of independently arisen cytological mechanisms for gamete production, which range from completely ameiotic processes (apomixis) to those involving more or less distorted meiotic divisions (automixis) (Stenberg & Saura, 2009, 2013). Consequently, some obligate asexuals transmit their genomes in a strictly *clonal* way (Glossary) while others, like hybridogens, transmit clonally only parts of their genomes (Figure 1). In fact, even the most prominent example of ancient asexuals, the bdelloid rotifers, show signatures of recombination between homologous chromosomes and occasional gene exchange between conspecifics (Debortoli et al., 2016; Signorovitch et al., 2015; Simion et al., 2021). The distinction between sexual and asexual reproductive modes is particularly difficult in plants, where various intermediates exist and several types of apomixis may emerge from one type of sexuality (Hojsgaard & Hörandl, 2015).

Variability also exists with respect to the process of fertilization. True parthenogens (Figure 1a) are typically females that form a new generation of daughters from unreduced eggs and thus are completely independent of gamete fusion. We use the term female only to indicate that these individuals naturally produce eggs, a trait they share with sexual females. Other types of asexuals are referred to as *sperm‐dependent parthenogens* (sometimes also as *pseudogams* or *sexual parasites*; Glossary) as they rely on sperm, which is usually, but not always, provided by closely related sexual species (e.g., Choleva et al., 2008; Schlupp, 2005). Gynogenetic females (*gynogenesis*; Glossary and Figure 1b) need the sperm just for egg activation with its genome excluded immediately after fertilization. They produce unreduced eggs that are activated by sperm, but the sperm genome does not contribute to the next generation of daughters that are genetically identical to their mother. In rare cases, the sperm may also contribute genetically to the otherwise clonal progeny by subgenomic components, e.g., microchromosomes (Figure 2a; Schartl et al., 1995), or by the incorporation of its entire genome which leads to a ploidy increase (genome addition; Figure 2b,f). Hybridogenetic pseudogams (*hybridogenesis*; Glossary and Figure 1c), typically females, but in some cases also males, eliminate all chromosomes of one parent (typically the male) during gametogenesis and produce gametes with the clonal genome of the other parent. A hybridogenetic female producing haploid clonal gametes then mates again with a male, resulting in a new generation of hybridogens. Since only half of the genome is clonally transmitted from generation to generation, hybridogenetic reproduction is referred to as hemiclonal. In some instances, like in the case of *Ambystoma* salamanders, repeated rounds of hemiclonal reproduction and genome incorporations have led to the replacement of the original parental genomes in hybrid lineages (the mode of reproduction known as *kleptogenesis*; Glossary; Bi et al., 2008; Bogart, 2019).

Because of their atypical meiosis and non‐Mendelian propagation of genomes, obligate asexuals serve as excellent models for understanding fundamental questions in evolution, ecology, and cell biology (e.g., Bengtsson, 2009; Brockhurst et al., 2014; Dalziel et al., 2020; Laskowski et al., 2019; Lively & Morran, 2014; Meirmans, 2009; Van Valen, 1973). Although seemingly rare compared to sexuals, they evolved independently in different taxonomic groups of metazoans (Fyon et al., 2023), suggesting this trait has high relevance for evolution and represents a fascinating challenge to what we understand as “typical” reproductive mode and sex.

In this review, we do not intend to provide an exhaustive overview of all asexual taxa, which would be a book‐length endeavor, nor to summarize all research on asexual organisms, which recently attracted new critical review and synthesis (e.g., Fujita et al., 2020; Laskowski et al., 2019) and contributed significantly to understanding the disadvantages and advantages of meiotic sex. Instead, we point out that ongoing research on traditional questions relating to asexuality often lacks careful consideration of the effects—both negative and positive—that asexuals have on the sexually reproducing species they occur with. This is especially the case of sperm‐dependent parthenogens, which are the main focus of our review. Sperm‐dependent parthenogens were traditionally viewed as combining disadvantages of both reproductive modes, asexuality and sexuality because they are deprived of regular recombination and segregation of their genetic material, while on the other hand, they cannot take full advantage of asexual reproduction, being dependent on mating with sexual counterparts (reviewed in Beukeboom & Vrijenhoek, 1998). To counterbalance this view, here we compile evidence for wide‐ranging effects that sperm‐dependent asexuals have by interacting with sexual species they coexist with. For example, by playing a role as sexual parasites that “steal” gametes of sexual species for their own reproduction (Avise, 2008; Hubbs, 1964; Lehtonen et al., 2013), they can indirectly affect the population dynamics of their sexual counterparts and negatively impact their *effective population size* (*N*_e_; Glossary) and carrying capacity, thereby modifying their gene pool. Overlooking or ignoring such aspects of evolution and ecology of sperm‐dependent asexuals may lead to an incomplete understanding of ecological and evolutionary dynamics in sexual–asexual populations.

Based on recent advances, we show that the very existence of sperm‐dependent asexuals, in general, implies several effects on populations and ecosystems they are embedded in and very important properties of coexisting sexual species in terms of their (1) genetic architecture, (2) diversification and speciation, and (3) spatial distribution. We also argue that these broad effects may last beyond the tenure of the individual asexual lineages causing them. Just as there is no clear line between sex and asexuality, we also note that some impacts on coexisting species are typical only for sperm‐dependent asexuals while some may be enforced by asexual reproduction in a broader sense. Therefore, even though in the following text we will focus primarily on the influence of sperm‐dependent parthenogens on sexual species, some examples will refer to asexuals in general. Box 1 describes three model taxa often mentioned throughout the text.

BOX 1 **Model systems.**

**Amazon molly (*Poecilia*)**

The Amazon molly (*Poecilia formosa*) is an all‐female, Live‐bearing fish of hybrid origin (Hubbs & Hubbs, 1932; Warren et al., 2018). It reproduces by sperm‐dependent parthenogenesis (gynogenesis; Schlupp, 2005; Figure 1b). Because Live‐bearing fishes have internal fertilization, Amazon molly females must copulate with males of another species to obtain sperm. The two main sperm donors, *P. latipinna* and *P. mexicana*, are also the parental species of this hybrid. Amazon mollies occur from the Rio Grande Valley (Southern Texas) to the Río Tuxpan (Northeastern Mexico; Schlupp et al., 2002). Amazon mollies were the first clonal vertebrate to be described (Hubbs & Hubbs, 1932). They have since been a model system for understanding the role of ecology, evolution, and behavior, especially mate choice in the maintenance of such asexual/sexual mating systems (Lampert & Schartl, 2008; Schlupp, 2009, 2010; Schlupp & Plath, 2005; Schlupp & Riesch, 2011).

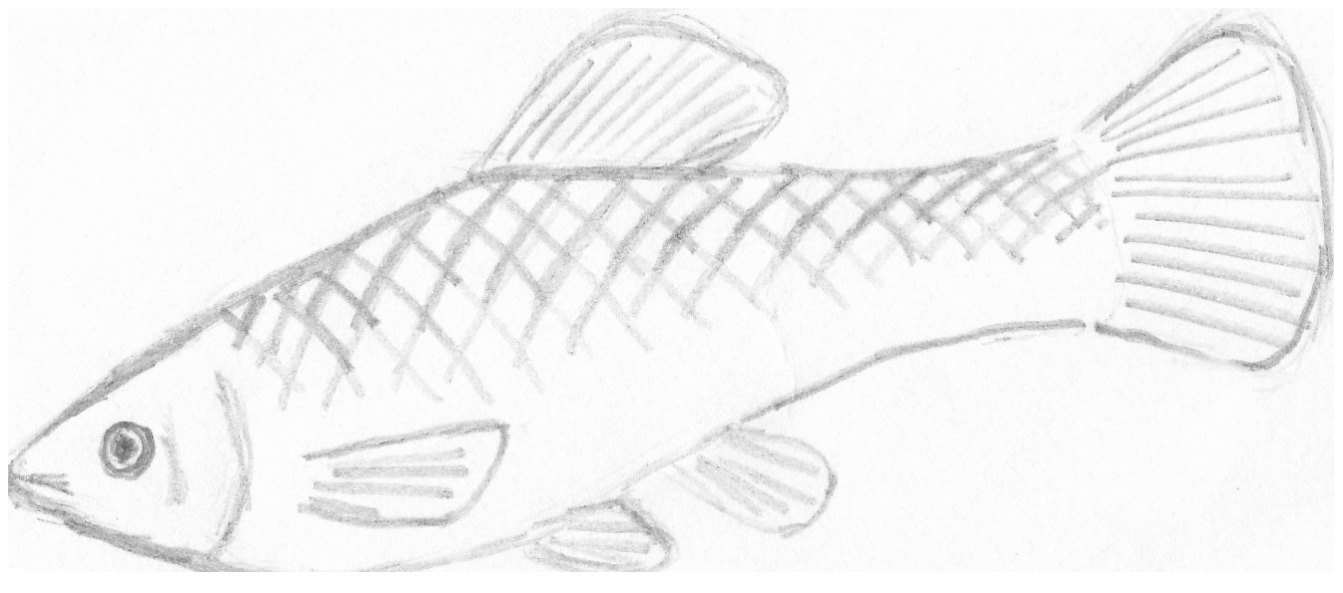

**Loaches (*Cobitis*)**

The so‐called *Cobitis taenia* hybrid complex comprises several sexually reproducing species of European freshwater fish which, during their diversification, got repeatedly into secondary contact and produced a wide spectrum of hybrids including sexual, sterile, polyploid as well as asexual forms (Janko et al., 2007). As the species diverge from each other, their hybrids gradually lose capability to produce reduced recombinant gametes while likelihood of hybrid asexuality has increased. Loaches, thus, demonstrated that hybrid sexuality may represent a primary speciation barrier between nascent species (Janko et al., 2018; Marta et al., 2023).

When parental species are at appropriate stage of genetic divergence, they may give rise to asexual hybrids very frequently (Janko et al., 2012) and although asexuality emerged repeatedly in different hybrid strains, its cytological background has been canalized into similar developmental pathways involving chromosomal endoreplication in gonadal cells (Dedukh et al., 2020; Kuroda et al., 2018). This ensures that during subsequent meiotic divisions, bivalents form between sister copies of chromosomes, thereby theoretically yielding no variability after crossovers. Interestingly, the capability of clonal gametogenesis is confined to hybrid females, while hybrid males are usually sterile due to aberrant pairing of orthologous chromosomes. Female asexuality, thus, not only represents a speciation barrier but also a remedy to hybrid sterility.

In contrast to expectations about long‐term disadvantages of asexuality, some *Cobitis* clones maintained high fitness for several hundreds of thousands of generations and established dominant components of many local populations (Kočí et al., 2020).

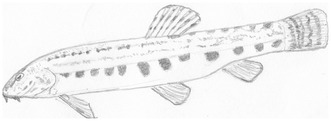

**Water frogs (*Pelophylax*)**

A central‐European complex of water frogs (genus *Pelophylax*) comprises parental species *P. ridibundus* (Marsh frog, RR) and *P. lessonae* (Pool frog, LL) whose hybridization gives rise to the hybridogenetic taxon *P. esculentus* (Edible frog, RL). In most populations, hybrids premeiotically eliminate *lessonae* (L) genome, produce gametes with hemiclonal *ridibundus* (R) genome, and form a new generation of hybrids by backcrossing with *P. lessonae* (Uzzell & Berger, 1975). A “mirror” system involves hybrids producing *lessonae* (L) gametes and mating with syntopic *P. ridibundus*. In the northern part of the range, hybrids live without the parental species forming all‐hybrid populations. Their perpetuation is achieved via mating between diploids and two types of triploids (LLR and LRR). Diploid hybrids form both clonal diploid (RL) and hemiclonal haploid (R) gametes. Triploid hybrids eliminate the parental genome present in a single copy and subsequently recombine the remaining two homospecific genomes producing recombined L and R gametes. Recombination and formation of different types of gametes liberate the hybridogens in all‐hybrid populations from the dependence on the parental species (Christiansen & Reyer, 2009; Christiansen et al., 2005). Hybridogenetic hybrids in this complex serve as vehicles for bidirectional but limited flow of nuclear genes between the parental species what motivated Uzzell et al. (1977) to name this complex as “leaky hybridogenetic”. Contrarily to limited bidirectional nuclear interspecific gene flow, mtDNA introgression is widespread and strictly unidirectional resulting in about 30% of *P. ridibundus* individuals in western and a part of central Europe possessing mitogenome of *P. lessonae* (Plötner et al., 2008).

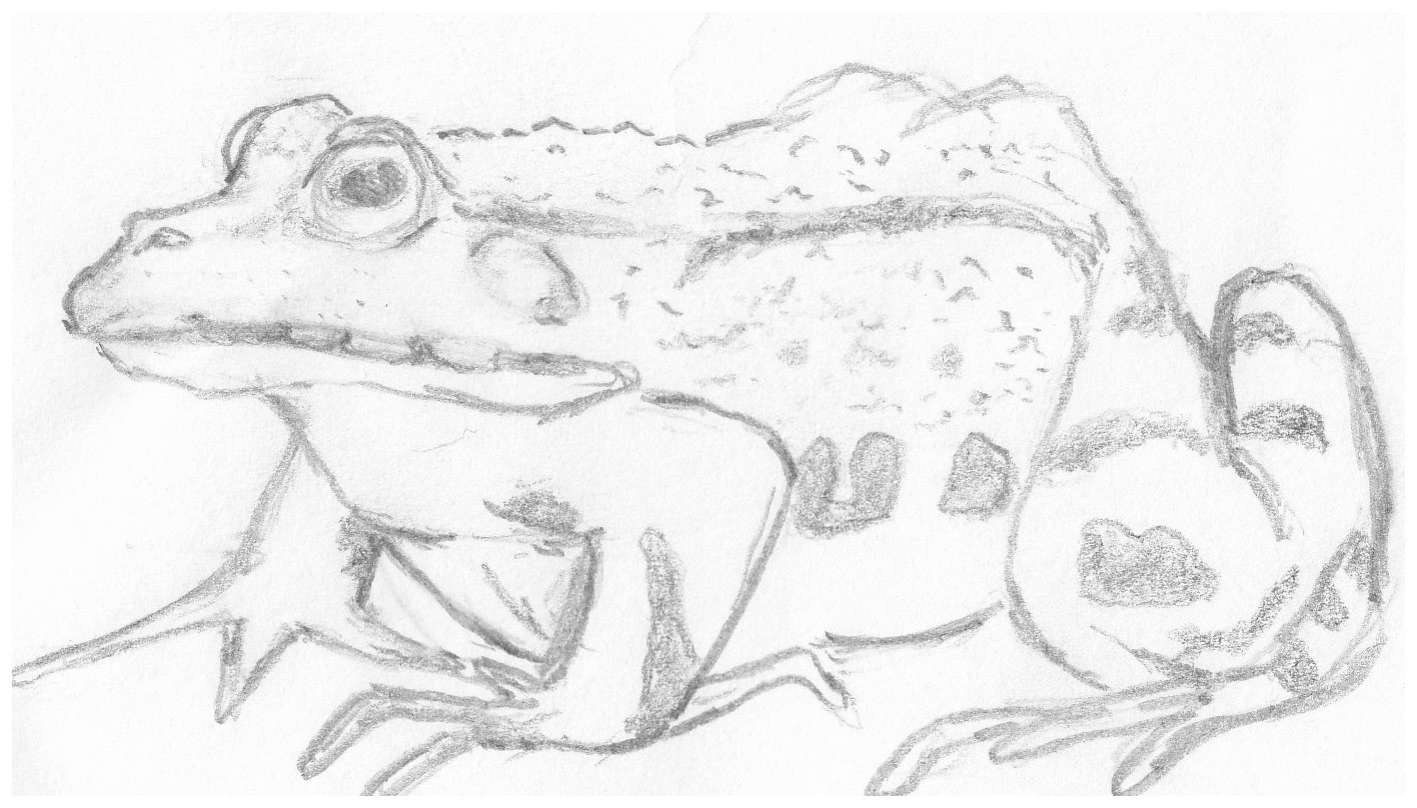

## 2 IMPACT ON THE GENE POOL OF SEXUAL SPECIES

### 2.1 Direct impact on a sexual species' gene pool via asexual‐to‐sexual gene flow

Asexual species are typically assumed to produce clonal progeny, but clonality is seldom perfect, allowing gene flow from the asexual to the sexual gene pool. In plants, for instance, phylogenetic reconstruction of angiosperm evolution indicated that reversals from apomixis to sexuality occurred (Hörandl & Hojsgaard, 2012). Backcrossing to sexual relatives has been directly reported from several apomictic species, e.g., in *Hieracium*, *Ranunculus*, and *Taraxacum* (Hörandl & Paun, 2007), which supposedly helps to generate new cytotypes (Sailer et al., 2020) and local reversal to sex (Majeský et al., 2012), thereby contradicting Darlington's “dead end of evolution” hypothesis (Darlington, 1939).

Here, we argue that asexual‐to‐sexual gene flow may be surprisingly common and effective, especially in systems with sperm‐dependent asexuals since they produce gamete types that enable their genes to flow back into sexual populations (Figure 2c–e) as was evidenced in various animal taxa ranging from flatworms to vertebrates (Angers et al., 2018; D'Souza & Michiels, 2009; Goddard & Schultz, 1993; Hotz et al., 1992; Scali, 2009; Sousa‐Santos et al., 2006; Vorburger, 2001). To understand this potential mechanism, let us first assume a typical hybridogenetic female hybrid between two species A and B which pre‐meiotically eliminate A‐type chromosomes, thus clonally transmitting only the B genome into her eggs. Normally, these eggs are fertilized by A‐sperm restoring the hybrid state of AB, with the A genome being recruited from a sexual population every generation, while the B‐derived genome is passed down asexually for many generations. However, when such a hybridogenetic female mates with a male of the B species, the fertilization with B‐sperm would lead to the formation of seemingly normal BB diploids, which reproduce sexually, but half of its nuclear genome has been introgressed from coexisting asexuals (see Figure 2c; Denton et al., 2018; Kwan et al., 2019; Mikulíček et al., 2014; Suzuki et al., 2019). As we explain later in this section, the fact that such BB individuals possess half a nuclear genome that has been evolving asexually for many generations may have considerable consequences as its evolutionary history likely differs from patterns expected under sexuality.

Furthermore, some sperm‐dependent parthenogens do not pass on their genomes only via females, but also involve males producing unreduced or hemiclonal sperm (e.g., *Pelophylax* frogs [Graf & Polls‐Pelaz, 1989; Mikulíček et al., 2015; Skierska et al., 2023]) or are hermaphrodites (e.g., *Schmidtea* flatworms [D'Souza & Michiels, 2010], or *Corbicula* clams [Hedtke & Hillis, 2011; Hedtke et al., 2008]) which fertilize related sexual females, thereby also facilitating the introgression of asexual genomes back into sexual gene pools. We note that similar instances of ‘contagious asexuality’ via males are also known from some obligate parthenogens, like *Daphnia* water fleas (Paland et al., 2005) or *Artemia* crustacean (Boyer et al., 2023).

Clearly, such gene flow from an asexual to a sexual gene pool fundamentally differs from any classical mechanisms of gene exchange between sexual species because asexuals transmit their genetic material *en bloc*, and in a non‐Mendelian fashion (Glémin et al., 2019). Since many asexuals are of hybrid origin and reproductively interact with their two parental species, they, thus, can serve as a hub between sexual species. However, owing to the specific nature of asexual transmission of genomes, the effect on the recipient genomes would be different from other types of introgressions found in ‘classical’ recombining sexual hybrids.

For example, if the aforementioned hybridogenetic hybrid originated from hybridization between a female from species A and a male from species B, its eggs would possess A‐type mtDNA but B‐type nucleus (Figure 2c). If fertilized by B‐sperm, such gametes would, thus, create cyto‐nuclear mosaics (also known as *cybrids*; Glossary) and hence facilitate the transfer of cytoplasmatic and/or complete nuclear genomes at rates superseding many unidirectional backcrosses via meiotic hybrids. Such massive unidirectional mtDNA flow mediated by sperm‐dependent parthenogens without any substantial nuclear admixis has been reported, for example, in water frogs *Pelophylax ridibundus* (Hotz et al., 1992; Mikulíček et al., 2014; Plötner et al., 2008), several species of the *Bacillus* insect (Scali, 2009), asexual *Corbicula* clams (Hedtke & Hillis, 2011; Hedtke et al., 2008) as well as various fishes including loaches of the family Cobitidae (Kwan et al., 2019), and cyprinids of the genera *Squalius* (Alves et al., 2001, 2002; Sousa‐Santos et al., 2006) and *Chrosomus* (Angers et al., 2018; Binet & Angers, 2005). Angers et al. (2018) documented that such a type of mtDNA introgression might have had considerable adaptive value during the postglacial range expansion in *Chrosomus*. In an extreme case, the gene flow from asexuals might have even caused complete replacement of the original mtDNA by an allospecific mitochondrial genome as suggested by cytonuclear mosaicism of *Cobitis tanaitica* spined loaches (Choleva et al., 2014). Sperm‐dependent asexuals may also mediate interspecific gene flow between nuclear gene pools of related sexual species, as found in the European complex of water frogs where hybridogenetic hybrids *P. esculentus*. Here, occasional recombination between parental genomes in hybridogenetic hybrids leads to introgression of (sexual or “fresh”) *lessonae* alleles into (asexual) *ridibundus* chromosomes that have been passed on hemiclonaly. This process was not only assumed to “rejuvenate” the asexually transmitted genome but also the backcrossing of such hybridogens to parental species may subsequently have mediated introgression of nuclear alleles between parental species *P. ridibundus* and *P. lessonae* (Mikulíček et al., 2014; Schmeller et al., 2005; Uzzell et al., 1977).

Another noteworthy, albeit hypothetical, aspect of such asexual to sexual introgression is related to the possibility that non‐Mendelian heredity of asexuals not only modifies the ways how their genome may be introgressed into interacting populations, but it may specifically affect the evolution of such genetic elements. To appreciate such a possibility, let us note asexual genomic elements that introgress into a sexual gene pool have evolved many generations in a (quasi)clonal way and passed through very different selection regimes than homologous sequences in a sexual species' gene pool. For instance, asexual genomes are assumed to evolve under relaxed purifying selection due to whole‐genome linkage, suggesting that a recipient sexual species may acquire genetic material with high numbers of deleterious mutations, depending on the duration of clonal evolution and the speed of mutation accumulation processes, like *Muller's ratchet* (Glossary). Moreover, asexual species may be selected for complete loss or loss of function of genes, which code traits that are vital for sex, but not needed or even maladaptive for asexual reproduction, such as genes related to meiosis or mating behavior (Parker et al., 2019; Schartl et al., 1991; Schlupp et al., 1992; Stork et al., 2022; van der Kooi & Schwander, 2014). The effect of introgression of such alleles or genomic parts into sexual gene pool may, thus, substantially differ from classical interspecific gene flow. It would, therefore, be instructive to investigate how such introgression of asexual genomes may deteriorate the gene pool of the sexual recipient species beyond the simple effect of mutation accumulation.

The aforementioned examples showed that at least in some instances the reproductive interactions with sperm‐dependent asexuals have influenced the gene pool of their sexual hosts. Therefore, we suggest that it would be interesting to investigate how important a source of maladaptive alleles in sperm‐dependent asexuals may be compared to other interactions that affect sexual gene pool. Clearly, hybridization in the form of introgression with closely related species is common and may introduce maladaptive alleles even without any involvement of asexuals at all, as documented for human–Neanderthal–Denisovan gene flow (Sankararaman et al., 2014). However, in the case of sexual–asexual interactions, the “maladaptiveness” does not come from admixis between differently adapted gene pools but rather from introgression of deleterious alleles which accumulate in asexual genomes due to their specific selection regimes. Moreover, it is often the case that when a pair of sexual species is capable of producing asexual hybrids (either sperm‐dependent or true parthenogens), sexually reproducing hybrids usually do not occur there as they are either sterile or inviable (see Dedukh et al., 2021 and references therein). Nevertheless, predicting the negative impact of gene flow from asexuals into sexual gene pools is difficult as models for mutation accumulation in asexuals are far from clear. For example, mutation rates seem to be generally male‐biased, which might counteract Muller's ratchet (Redfield, 1994) and partly explain why accumulation of deleterious mutations appears to be surprisingly slow in many investigated asexual all‐female species (Janko et al., 2011; Kočí et al., 2020; Pellino et al., 2013; Warren et al., 2018). Furthermore, in Amazon mollies (*P. formosa*), a gynogenetic fish species from Southern Texas and Northeastern Mexico, loss of sex‐related traits was not found (Warren et al., 2018). By contrast, in fully asexual organisms such as the snail *Potamopyrgus antipodarum*, loss of male function has been reported (Jalinsky et al., 2020).

On the other hand, we suggest that introgression from asexuals may also be beneficial for sexual recipients since restriction of recombination may have some positive aspects for genome evolution. Especially, it may favor the spread of advantageous combinations of alleles in regions where recombinants are expected to have lower fitness (Neiman & Linksvayer, 2006), similar to the evolution of ‘supergenes’ (Thompson & Jiggins, 2014). Selection for restricted recombination has indeed been documented between loci contributing to adaptation (Thompson & Jiggins, 2014), speciation (Ortiz‐Barrientos et al., 2016), or de novo evolution of separate sexes (Charlesworth & Charlesworth, 1978). Asexual gene pools, for their whole‐genome linkage, may, therefore, represent unprecedented testing fields where selection acts on various allelic combinations with much greater efficiency than could ever be observed in any sexual species, and multiply, due to clonal reproduction, the fittest multilocus genotypes (Barbuti et al., 2012). Therefore, when asexual‐to‐sexual gene flow is possible, it is likely to introduce whole chromosomes that evolved for a long time without recombination into particularly suitable combinations of alleles. Detecting such positive effects of gene exchange with asexual hybrids is challenging, but for instance, Schmeller et al. (2005) proposed that bidirectional introgression between sexual water frog species *P. ridibundus* and *P. perezi* mediated by the hybridogenetic hybrids *P. grafi* increased the probability of local adaptation to hypoxic conditions and range expansion of the sexual species.

### 2.2 Indirect impact on a sexual species gene pool via modification of effective population size

Yet, even without direct gene flow from an asexual species, the gene pool of sexual species would still be modified indirectly by interactions with coexisting sperm‐dependent asexuals, because the very presence of those likely affects selective pressures operating in sexual populations and their demographic parameters. To understand the underlying reasons, it must be kept in mind that although asexual taxa often diverged ecologically from their sexual counterparts (see e.g., Ross et al., 2012; Van der Kooi et al., 2017), many asexual species, especially the sperm‐dependent parthenogens, are ecologically relatively similar to their sexual ancestors, with which they often coexist (Beukeboom & Vrijenhoek, 1998; Vrijenhoek & Parker, 2009). Consequently, part of the environmental carrying capacity potentially available for the sexual population is taken up by asexuals when these are present and in fact, the sperm‐dependent asexuals often vastly outnumber their sexual hosts so that their proportions in mixed populations reach over 70% or more, (e.g., in frogs [*Pelophylax*; Graf & Polls‐Pelaz, 1989; Mikulíček et al., 2015]), salamanders (*Ambystoma* [Bogart et al., 2009]), and fishes (*Squalius* [Cunha et al., 2008]; *Cobitis* [Janko et al., 2007]; and *Poecilia* [Heubel & Schlupp, 2008]). Hence, the impact of their presence is worth being considered.

**FIGURE 3:**
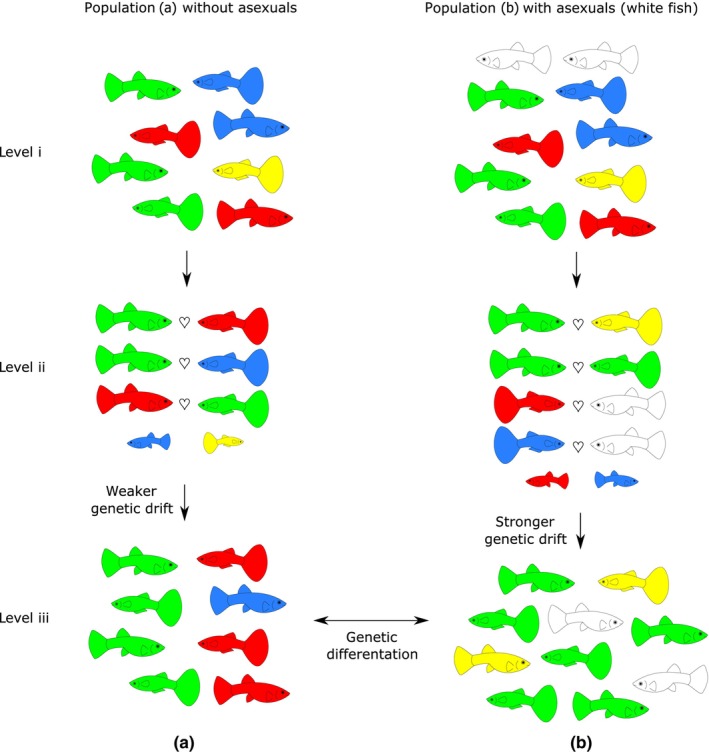
The effect of coexisting sperm‐dependent parthenogens on sexual host's effective population size and related phenomena: Compared to a purely sexual population (a), when a sexual population of the same census size coexists with sperm‐dependent parthenogens (b; level i), it suffers from increased variance in reproductive success since relatively fewer males have access to conspecific sexual females and ‘waste’ their reproductive effort on asexual females (level ii). This negatively affects the long‐term effective size of the sexual population (level iii). Such intensification of genetic drift also leads to faster population differentiation. Effect on operational sex ratio (OSR) and mate choice: Because males are in a minority when coexisting with a sperm‐dependent all‐female population, it changes the OSR. Simultaneously, given that mating with sperm‐dependent females effectively ‘wastes’ males' reproductive effort, an intensified selection for mating preferences with conspecific is expected. Colored fish represent sexually reproducing individuals, white fish are sperm‐dependent parthenogens. Colored fish of a smaller size without a heart mark between them (level ii) do not reproduce in the population, which is reflected in reduced genetic variability in the next generation (level iii).

In general, there are several mechanisms how sperm‐dependent competitors may affect the effective population size (*N*_e_; Glossary) of related sexuals. These include stochastic effects increasing the strength of genetic drift, such as (a) modifying extinction/recolonization dynamics of sexual metapopulation, (b) increasing the variance in reproductive success of the sexual species, and (c) modifying selective pressures resulting from biased operational sex ratio (OSR) in mixed sexual–asexual populations. Let us consider these mechanisms in the following paragraphs:

- Sperm‐dependent parthenogenesis depends on access to males of a sexual species and consequently the asexuals may not outcompete their sexual host, lest they lose a vital resource, leading to the collapse of the whole sexual–asexual complex (Schlupp & Riesch, 2011). Kokko et al. (2008) published a mathematical model proposing that coexistence with sexuals is possible provided that outcompeting the sexual species by the asexuals occurs locally and asynchronously in discrete populations. Sexuals immigrating from nearby populations may re‐colonize the areas of extinction until being invaded by another wave of asexuals in a multi‐species metapopulation dynamic. For the sexual species, however, Kokko et al.'s model implies one additional consequence which stems from classical population genetic theory: if the sexual metapopulation is forced to pass through such extinction–recolonization cycles induced by sperm‐dependent parthenogens, it follows that its effective population size will be diminished. Additionally, it also follows that at each moment, some parts of range potentially suited for the sexual species will be temporarily unavailable.
- Coexistence with sperm‐dependent parthenogens also likely increases the variance in reproductive success in a host sexual species because sexual individuals may spend a considerable portion of their reproductive potential on mating with sperm‐dependent parthenogens rather than conspecific individuals (Schlupp, 2010). Consequently, in each generation, the sexual gene pool would be reconstituted from fewer fathers than it would normally have in absence of sperm‐dependent parthenogenetic females. It follows from classical population genetic theory that such an increased variance in reproductive success would subsequently further reduce the effective population size of the sexuals, especially in situations when the proportion of sperm‐dependent asexuals is high in mixed sexual–asexual populations (see above) (Figure 3).
- Another consequence of the presence of sperm‐dependent parthenogenetic females is that male mate choice as well as female competition (Makowicz & Schlupp, 2015) can occur because the operational sex ratio is female biased (Schlupp, 2009). Under some conditions, the OSR, the ratio of reproductively active males and females, may be altered, which has consequences for which sex is choosier (Amundsen, 2018; Schlupp, 2021). Simply put, if males become the rare sex, they switch from male competition to male choice. Such a mechanism has, for example, been documented in some sexual fishes such as two‐spotted goby, *Gobiusculus flavescens* (Forsgren et al., 2004). In this mating system, males become rare late in the season, and consequently become choosier than females showing how important ecological conditions can be in modifying sexual selection (Amundsen, 2018). The presence of large numbers of asexual females may have drastic effect on sex allocation and ratio in interacting sexual species. This occurs, for example, in sexual brine shrimp *Artemia franciscana*, which adaptively adjust their sex ratio under natural conditions. However, when co‐occurring with the related obligate asexual all‐female species *A. parthenogenetica* in recently invaded parts of its distribution range *A. franciscana* maladaptively produces extremely male‐biased sex ratio (Lievens et al., 2016). The presence of sperm‐dependent females in sexual/asexual mating systems may have an even stronger effect and alter the OSR in such a way that female choice is diminished, and male choice prevails (Figure 3). A skewed OSR and increased variance in reproductive success of a sexual species thus further reduces its effective population size, especially in those mixed populations where the proportion of sperm‐dependent parthenogenetic females is high. As we said above, such high proportions of sperm‐dependent parthenogens have been documented in several asexual–sexual complexes of frogs (*Pelophylax* [Graf & Polls‐Pelaz, 1989; Mikulíček et al., 2015]), salamanders (*Ambystoma* [Bogart et al., 2009]), and fishes (*Squalius* [Cunha et al., 2008]; *Cobitis* [Janko et al., 2007]; and *Poecilia* [Heubel & Schlupp, 2008]).

The coexistence with sperm‐dependent parthenogens, thus, seems to exert a systematic impact on reducing the effective population size of their sperm donors. The magnitude of such an effect indeed depends on the proportions of sexual to asexual individuals in mixed populations. Also, note that sex ratios are affected as almost all sperm‐dependent parthenogens are all‐female. Consequently, other mechanisms, such as mate choice, indeed require more attention aided by population genetic theory. For example, populations with diminished effective size have higher likelihood of inbreeding depression and extinction (Byers & Waller, 1999). Selection becomes less effective while the effect of genetic drift increases, allowing more frequent fixation of deleterious alleles due to chance. Additionally, the faster fixation of positively selected alleles leads to a greater loss of genetic diversity in small populations (e.g., Lacy, 1987; Lande, 1976). Population size reduction further increases the *Allee effect* (Glossary) in sexual populations and directly impacts metapopulation connectivity by reducing the effective number of migrants (Lowe & Allendorf, 2010). Consequently, lower efficiency of homogenizing geneflow speeds up local fixation of alternative alleles in small isolated populations (Cosentino et al., 2012), which may also contribute to faster adaptation to local environments, potentially limiting the plasticity of population‐wide responses to stochastic events.

Despite clear predictions, the detection of such indirect effects on sexual gene pools received little attention to date. Long‐term studies of sperm‐dependent asexual complexes may provide suitable model systems to test this hypothesis, however. Patterns in line with such predictions have already been noticed in some instances. For instance, we can assume that in a hybridogenetic complex of water frogs, the parental species *P. lessonae* is more affected by hybridogenetic hybrids *P. esculentus* because in most populations it serves as a gamete donor for them. Hybridogenetic hybrids, thus, could decrease the effective population size of *P. lessonae*. One of the consequences of reduced population size and the higher rate of genetic drift could be the higher genetic differentiation of *P. lessonae* populations compared to another parental species *P. ridibundus* that coexists less frequently with hybridogenetic hybrids and less frequently serves as a host species for them (e.g., Pruvost et al., 2015). Another example of such indirect effects comes from spined loaches, the *Cobitis* hybrid complex. Here, the gametogenic performance of males of the sexual species changed in response to a female‐biased sex ratio a in mixed sexual–asexual population (Jablonska et al., 2020; Juchno & Boroń, 2006). Specifically, unlike their counterparts from purely sexual populations, sexual males serving as sperm donors in mixed populations had to meet the reproductive challenge of a high proportion of sperm‐dependent parthenogenetic females, which led to continual year‐round sperm production, higher production of spermatogonia during and after spawning and lower rates of apoptosis in their testes.

## 3 IMPACT ON SPECIATION

In this section, we discuss how asexual organisms, and sperm‐dependent asexuals in particular, contribute to the existing biodiversity either indirectly by facilitating the speciation in coexisting sexual species or directly by forming new species themselves.

### 3.1 Indirect impact on speciation: promotion of reproductive isolation barriers

We first present several ways, how sperm‐dependent parthenogens, by their very presence, may affect population divergence as well as establishment of both prezygotic and postzygotic reproductive isolation barriers (RIB) between populations of related sexuals. Such mechanisms are rather cryptic and have become appreciated only recently.

*Postzygotic RIB*: As already mentioned, the presence of sperm‐dependent parthenogens can negatively impact effective population sizes and connectivity among demes of interacting sexual species. In effect, this mechanism ultimately increases local drift within sexual demes, and in turn, contributes to greater inter‐population differentiation among sexual demes (Figure 3). Moreover, if there is introgression from clones, local sexual populations may diverge from each other even more rapidly because introgression patterns mediated by asexuals substantially differ from those mediated by sexual hybrids (see Section 1).

In summary, the coexistence with sperm‐dependent asexuals is expected to affect the connectivity among demes of their sexual host species, thereby increasing its genetic fragmentation and potentially enhance its rates of the allo‐/or peripatric speciation. Of course, there are multiple mechanisms affecting the connectivity among demes of any sexual species, but coexistence with sperm‐dependent asexuals leads to systematic pressure toward genetic fragmentation, calling for greater focus on this type of interaction.

Moreover, asexual hybrids can themselves play a role as primary postzygotic barrier and hence promote speciation between diverging taxa. Indeed, postzygotic reproductive incompatibilities tend to accumulate with genetic divergence between emerging species (Seehausen et al., 2014), and their initial stages are generally characterized by decreased fertility or sterility of hybrids, while hybrid viability tends to be compromised only at later stages with substantial genetic divergence (Bolnick & Near, 2005; Edmands, 2002; Matute et al., 2010; Russell, 2003). Interestingly, the likelihood of asexual reproduction in hybrids also appears to correlate with divergence between parental species, following a continuum from sexually reproducing hybrids between closely related parents to obligate asexual hybrids between distant parental species (reviewed in Janko et al., 2018; Stöck et al., 2021). This trend has been noted over a century ago by Ernst (1918) and it has been later suggested that distortion of hybrid's gametogenesis towards production of clonal gametes is possible in a particular ‘window of genetic divergence’ before complete hybrid sterility emerges (Carman, 1997; De Storme & Mason, 2014; Moritz et al., 1989; Wetherington et al., 1987). In some instances, the formation of hybrid asexuality has even been shown to share a common cytogenetic background with hybrid sterility (Dedukh et al., 2020; Janko et al., 2018; Marta et al., 2023).

**FIGURE 4:**
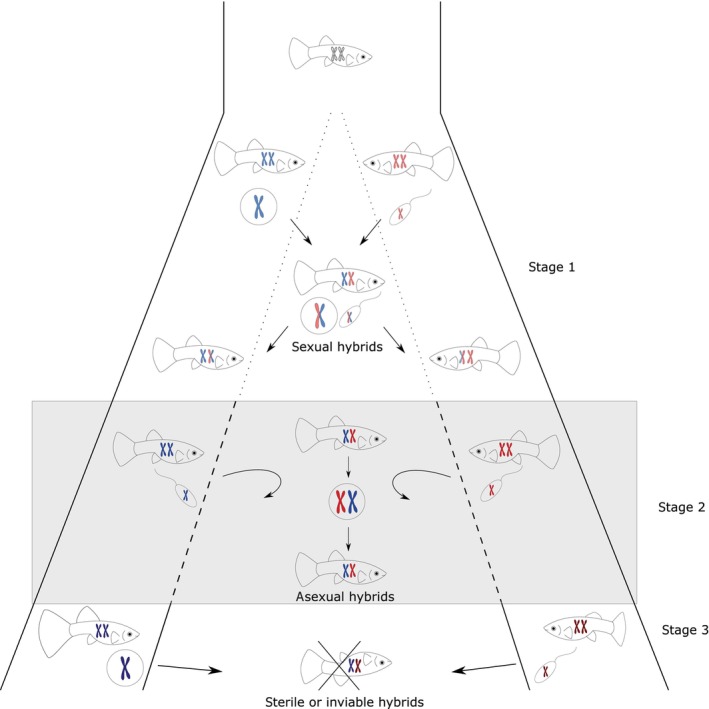
The classical scenario of postzygotic reproductive isolation assumes that as nascent species diverge (stage 1 in the scheme), their genomes (denoted as bicolored chromosomes) become progressively less compatible and interspecies barriers become more pronounced (as denoted by dotted to solid lines) until a stage is reached when species cannot produce fertile or viable hybrids and speciation is complete (stage 3 in the scheme). Hybridizing species in stage 1 form sexual hybrids, in stage 2 asexual hybrids, and in stage 3 hybrids are sterile or inviable (crossed upside‐down fish). The ability to produce asexually reproducing hybrids probably also scales with genetic divergence between hybridizing taxa (Moritz et al., 1989). Empirical data (Janko et al., 2018) suggest that such a phase (stage 2 in our scheme) may occur generally at earlier stages of species differentiation before complete hybrid sterility occurs (stage 3). If so, asexual hybrids, although as fertile as “classical” sexual hybrids produced at early stages (stage 1 in the scheme), may turn into an effective barrier to interspecific gene flow due to their general inability to backcross into either parental species.

Thus, a century after seminal works by Bateson (1909) and Ernst (1918), it is becoming clear that the formation of hybrid asexuality has many analogies with Bateson–Dobzhansky–Muller speciation models. From this perspective, asexual reproduction in hybrids may be viewed as a special type of postzygotic incompatibility where on one hand, the production of clonal gametes stems from accumulated incompatibilities in cell cycle regulation between parental species (e.g. Carman, 1997; Moritz et al., 1989), while on the other hand the unrecombined gametes restrict the gene flow between parental species imposing an effective reproductive barrier among hybridizing species (Janko et al., 2018; Lampert & Schartl, 2008). Comparative analysis of reproductive modes in vertebrate hybrids showed that asexually reproducing hybrids generally tend to emerge at lower interparental divergences than completely sterile or inviable hybrids, (Janko et al., 2018; Stöck et al., 2021), suggesting that asexuality‐related barriers may arise at earlier stages of speciation than other barriers like complete hybrid sterility or inviability (Figure 4). Empirical examples do support the view of a link between the ‘classical speciation continuum’ and hybrid asexuality (Janko et al., 2018; Lampert et al., 2007), which has recently been called ‘extended speciation continuum’ (Glossary; Stöck et al., 2021). Among vertebrates, for instance, on one end, there are dynamically hybridizing species pairs that produce diverse assemblages of asexual hybrids such as *Cobitis* (Choleva et al., 2012), *Pelophylax* (Hoffmann et al., 2015; Hotz et al., 1985), and *Poeciliopsis* (Schultz, 1973). On the other end, there are clonally reproducing hybrid taxa that stem from a single historical hybridization event and attempts to cross their extant sexual ancestors fail to produce clones but lead to sexual F_1_'s, such as *Poecilia* (Lampert & Schartl, 2008; Stöck et al., 2010). Cases like the fish *Chrosomus* (the former *Phoxinus*) may represent an intermediate condition, where phylogenetic analysis indicated polyphyletic origins of diverse asexual assemblages but new clones can no longer be produced by hybridization among contemporary parental species, possibly because they are already too diverged to produce fertile hybrids of any sort (Angers & Schlosser, 2007). Some species pairs may also give rise to both, the clonal and sexual hybrids, like, for example, fishes *Fundulus* (Hernández Chávez & Turgeon, 2007) and *Rutilus rutilus* × *Abramis bramma* (Slyn'ko, 2000).

Fyon et al. (2023) suggested that the establishment of a sperm‐dependent parthenogen may be more complex and numerous fundamental traits may evolve gradually. For instance, while the ability to produce unreduced gametes may emerge “instantaneously” as a result of hampered crosstalk between diverged parental genomes (Marta et al., 2023), others, like the ability to reject sperm's genome after fertilization, may evolve subsequently. Nevertheless, theoretical models suggest that once a successful sperm‐dependent asexual hybrid forms an established clonal lineage, it is likely to numerically overwhelm its hosts in hybrid zones, and consequently further reduce the reproductive contacts between parental species (Janko et al., 2019). This would further promote the formation of interspecific reproductive barriers because the prevalence of sperm‐dependent parthenogens in such hybrid zones would additionally restrict any mating between parental species, thereby preventing possible introgression (Janko et al., 2019).

The link between speciation and asexuality (Janko et al., 2018; Stöck et al., 2021) has important implications for the perception of the role of asexuality in evolutionary biology. Asexual lineages are presumably short lived on an evolutionary timescale (Butlin et al., 1999), and given that the phase when diverging species can produce asexual hybrids is transient, the likelihood of detecting natural clones of hybrid origin is, therefore, limited to hybrid zones between sexual species in a ‘proper’—and presumably short—stage of divergence. It is, therefore, possible that various extant “good” sexual species might have historically produced asexual hybrids as part of the speciation process, but transitory asexual forms are now extinct. Contemporary research on asexuality generally focuses on naturally occurring clones, which, by definition, constitute only a subset of evolutionarily successful and stable lineages. It would, therefore, be appealing to focus more on potential existence of asexuality among experimental progenies of sexually reproducing species, which may test the hypothesis of the extended speciation continuum.

*Prezygotic barriers*: The presence of sperm‐dependent parthenogens also affects the premating isolation mechanisms, for example, by exerting selection pressure on mate‐recognition systems in co‐occurring sexuals. While such asexuals rely on sperm, from the point of view of a sexual host mating with them represents a costly behavior and hence it has been postulated that the stability of sexual‐sperm‐dependent parthenogen complexes relies on the ability of sexual males to discriminate hybrid and conspecific females (Mee & Otto, 2010; Morgado‐Santos et al., 2015; Schlupp & Plath, 2005). This process is somewhat similar to reinforcement where the evolution of mate choice is often selected for in zones where ranges of hybridizing species overlap (Marshall et al., 2002) and character displacement is predicted (Gabor & Ryan, 2001). However, contrary to classical cases, the distribution of sperm‐dependent parthenogenetic hybrids represents something like a hybrid zone extended in time and space because such hybrids often expand over large parts of parental ranges, sometimes well beyond their area of origin (Janko et al., 2019). Hence, contrary to a classical reinforcement scenario which takes place in narrow zones of sympatry, sperm‐dependent parthenogens exert selective pressures over vast areas deep in allopatry and may, therefore, considerably speed‐up the establishment of prezygotic isolation.

While only a few studies examined geographical variation in male mate choice against asexuals, some empirical support for such hypothesis exists. For instance, Gabor and Ryan (2001) and Gabor et al. (2005) found that males of the sexual sailfin molly (*P. latipinna*) living sympatrically with gynogenetic Amazon mollies (*P. formosa*) showed a significantly stronger mating preference for conspecific females than males from populations that were allopatric with Amazon mollies. Another study by Gabor et al. (2013), however, showed that male mate choice varied geographically and may be associated with variation among populations in the length of sympatry with the gynogenetic Amazon molly (*P. formosa*). Furthermore, metapopulation dynamics may depend also on a conflict between species and mate quality recognition cues (visual, chemical, and tactile). Asexual–sexual mating systems in mollies represent complex networks where, moreover, not just male mate choice should evolve, but also interspecific female competition (Makowicz & Schlupp, 2015).

In addition, given that at least some sexual and asexual species pairs substantially differ ecologically (Pantel et al., 2011; Ross et al., 2012; Van der Kooi et al., 2017; Vrijenhoek, 1994), it may also be hypothesized that asexuals could drive character displacement in sexual species also in its ecological characters. In such a case, the presence of asexuals may ultimately generate ecological divergence between allopatric sexual populations and those in sympatry with asexuals, perhaps even providing a first step in ecological speciation.

### 3.2 Direct impact on speciation: species formation by asexuals

Finally, there is a more direct way how asexuals may contribute to biodiversity; they may form new species themselves. The question whether speciation can occur without sex is longstanding (Coyne & Orr, 2004; Dubois, 2011; Hausdorf, 2011; Shcherbakov, 2010) but recent theoretical and empirical studies show that species‐level taxa with distinct genetic, morphological, and ecological features may be formed also in asexuals (e.g., Birky & Barraclough, 2009; Carman et al., 2019; Cohan, 2001, 2002; Domes et al., 2007; Fontaneto et al., 2007; Franklin, 2007; Schön et al., 2012). The potential of diversification in asexuals is, thus, becoming increasingly appreciated.

However, asexuals may not only form species themselves but, as suggested especially in the botanical literature, they could play a role as stepping‐stones in the evolution of new sexual species, when reverting to sex. This process has been particularly accentuated as a possible explanation for the origin of hybrid and polyploid species (Figure 2f), where sperm‐dependent asexuals have been assumed to play an important role. Namely, because the emergence of a novel hybrid/polyploid form is supposedly a rare phenomenon and its establishment is, thus, threatened by a frequency‐dependent disadvantage (i.e., the minority cytotype exclusion principle; e.g., Husband, 2000), the establishment of novel strains could be facilitated by asexual reproduction, which offers immediate reproductive isolation and clonal multiplication of successful genotypes (Choleva & Janko, 2013; Hojsgaard & Hörandl, 2015; Rieseberg & Willis, 2007). Asexuality can, thus, represent the first stages toward hybrid speciation. For example, clonally reproducing triploids were suggested to serve as ‘triploid bridge’ toward tetraploid species with re‐assumed sexual reproduction (Choleva & Janko, 2013; Cunha et al., 2008; Dubey et al., 2019; Hojsgaard & Hörandl, 2015). This mechanism is particularly appealing in sperm‐dependent asexuals since they rely on sperm source and hence seem particularly prone to fertilization and subsequent increase in ploidy.

Recent data offer controversial support for this hypothesis since most known tetraploids derived from extant triploids are rather sterile or have a fitness disadvantage (reviewed in Choleva & Janko, 2013). Nevertheless, there exists empirical evidence that established obligatory asexuals may revert to sex, as found, for example, in Oribatid mites (Domes et al., 2007) and some plant taxa, like *Hieracium pilosella* (Fehrer et al., 2005). In fish, *Squalius* (Cunha et al., 2008), the asexuals relying on sperm apparently reverted to normal meiosis after gaining balanced genome composition following sperm incorporation.

It remains unclear why reversal to sex is not more common unless it often goes undetected. The apparent paucity of asexual‐to‐sexual transitions may result from the fact that established asexuals may be selected for the loss of sexual traits, which are disadvantageous or unnecessary for clonal reproduction (van der Kooi & Schwander, 2014), thereby preventing the re‐evolution of sex. However, this should not be the case in sperm‐dependent parthenogens, which have to maintain the full genetic machinery allowing them to mate with sexuals (Schlupp et al., 1998; Warren et al., 2018). Scarcity of asexual‐to‐sexual transitions may, thus, be only apparent, because the identification of sexually reproducing species which passed through a phase of clonality is extremely difficult, and probably easily overlooked.

There is indeed sound evidence for the ability of some sperm‐dependent parthenogens to form populations independent of sexual sperm donors, which might be a first step toward either true parthenogenesis or sexuality. Such cases have been recently documented in gynogenetic lineages of a nematode (Grosmaire et al., 2019) and sexual speciation in *statu nascenti* occurs in hybridogenetic water frogs, where a transition occurred from hemiclonal to sexual hybrids, which are reproductively independent of the parental species and form pure‐hybrid populations with a high proportion of triploids. Triploid hybrids form gametes with the genome of each of the parental species and thus substitute parental individuals in pure‐hybrid populations (Berger, 1983; Christiansen & Reyer, 2009; Christiansen et al., 2005; Figure 2b).

What makes such a case particularly interesting is the discovery by Stöck et al. (2002) who described an all‐triploid bisexual frog species, *Bufotes baturae*. This triploid bisexual species combines two genomes from distinct ancestors (two copies of the so‐called NOR^+^ genome and one copy of NOR^−^ genome), whose transmission to gametes sharply differs between males and females. While males eliminate the single NOR^−^ genome and recombine and segregate NOR^+^ genomes in order to produce haploid sperm, females produce diploid gametes containing clonally transmitted NOR^−^ genome and a recombined NOR^+^ genome. Fusion of such gametes restores triploidy in every generation. Given the similarity to the *P. esculentus* system, there is an intriguing possibility that bisexual *B. baturae* evolved through an asexual stage and that other systems, like *P. esculentus* may be on a similar evolutionary pathway just at a different stage.

These cases indicate that a transition from asexual to sexual is at least a plausible scenario for how asexuals can directly contribute to the formation of the regular sexual species.

## 4 IMPACT ON SPATIAL DISTRIBUTION OF SEXUAL SPECIES

**FIGURE 5:**
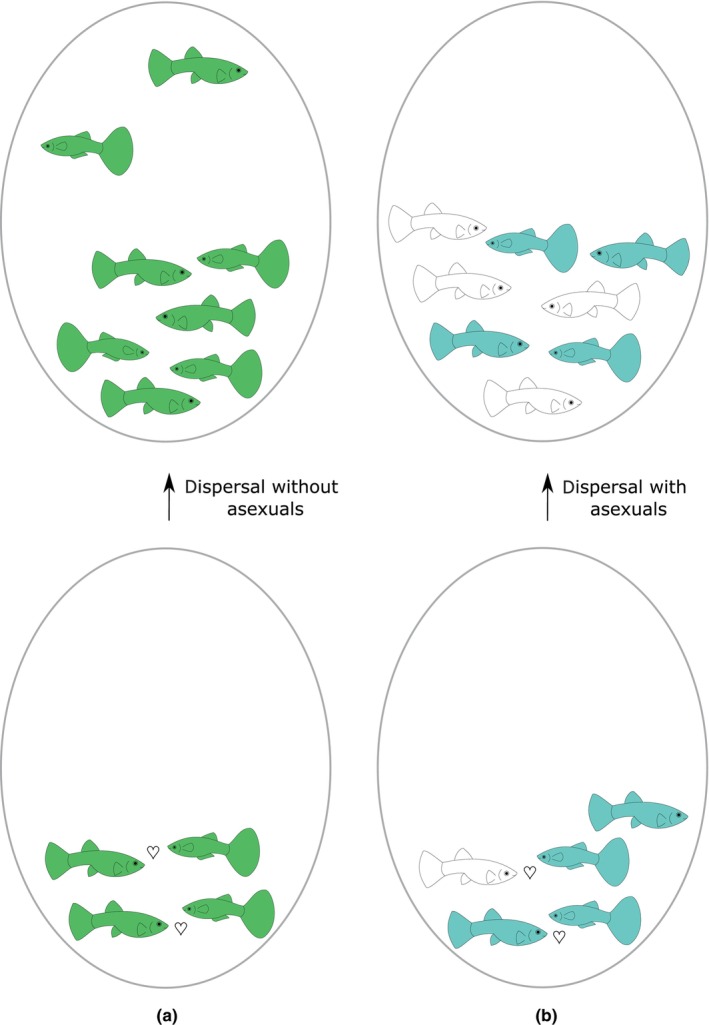
When sperm‐dependent parthenogens coexist with a sexual population (b), they tend to decrease its effective population size and therefore hamper the population growth rate. Expansions of sexuals to a new environment, thus, occur at lower frequency as compared to purely sexual populations (a) and therefore they have a lower probability of finding a proper mate to start a new generation of colonists. This ultimately decreases the growth rate and expansion rate of entire populations (Janko & Eisner, 2009).

The presence of sperm‐dependent parthenogens also has considerable demographic consequences for its sexual host whose population sizes and density are reduced by direct competition for males and other resources. Janko and Eisner (2009) used a mathematical model to demonstrate that a sexual population “infected” with sperm‐dependent parthenogens is expected to have a limited potential of spatial expansion and colonization of new habitats compared to a situation where no sperm‐dependent parthenogens affect it (Figure 5). Such a reduction in population expansion speed stems from the fact that sperm‐dependent parthenogens reduce the density of their sexual hosts along the expanding wave and therefore decrease their chance of finding a mating partner needed to establish viable populations in invaded areas. Hence, sperm‐dependent parthenogens may decisively affect large‐scale biogeographic patterns of their sexual hosts by delaying their spatial expansion into new habitats. Such effect may persist even after an eventual extinction of asexuals in case when different sexual species occupied these habitats in the meantime.

The biogeography of European loaches of the genus *Cobitis* potentially offers an empirical example of such a process. While in many European freshwater fishes the postglacial recolonization of Central and Western Europe proceeded from Danubian/Pannonian refugia, *Cobitis* fishes show a contrasting pattern since the Danubian species *C. elongatoides* experienced only a limited postglacial expansion reaching only the upper Odra and Elbe river watersheds, while most of Europe was colonized by *C. taenia* rapidly expanding from Eastern refugia (Janko et al., 2005). Interestingly, *C. elongatoides* populations survived the last glacial maximum in Danubian refugia together with sperm‐dependent parthenogenetic hybrids and co‐expanded with them into northern areas, which might have delayed its colonization rate as compared to *C. taenia*, whose expansion was not burdened by these sexual parasites (Janko et al., 2005).

**FIGURE 6:**
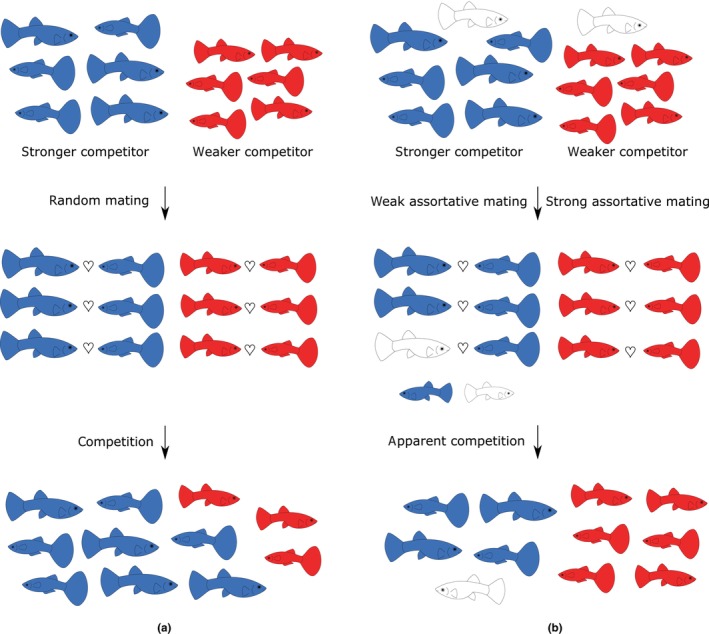
Compared to purely sexual competing species (a), when a stronger competitor (blue) outcompetes the weaker one (red), sperm‐dependent parthenogens may turn the result of interspecific competition (b). This occurs when the stronger sexual competitor invests more into mating with sperm‐dependent parthenogens (or is otherwise more vulnerable to their presence) than the weaker competitor, whose population growth is, therefore, faster and may ultimately outcompete the stronger one (Janko et al., 2019).

In addition, sperm‐dependent parthenogens may decisively affect the results of interspecific coexistence and competition among interacting sexual species. To appreciate this phenomenon, let us emphasize that many sperm‐dependent asexuals originated by hybridization between several sexual species (Choleva et al., 2012; Neaves & Baumann, 2011) and they can therefore simultaneously use (or parasitize) two or more sexual species for their own reproduction, (e.g., Choleva et al., 2008; Schlupp, 2005). In such cases, mathematical models of dispersal with competition showed that the sexual host species with better mate recognition ability or smaller niche overlap with coexisting parthenogens will be less negatively affected by their presence. Such a reduction of negative interactions with sperm‐dependent hybrid asexuals would give the host species an advantage in competition with other sexual species, whose demographic performance is harmed to a greater extent (Janko et al., 2019). Gynogens can, therefore, mediate the so‐called *apparent competition* (Glossary) among sexual species and cause an effect analogous to *parasite‐mediated competition* (Glossary; Holt & Bonsall, 2017; Thomas et al., 2000). In their presence, even a stronger sexual competitor may be outcompeted by a weaker one, if the latter is less negatively impacted by coexisting sperm‐dependent parthenogens (Figure 6). It follows that the effect on the diversity of sexual species would remain even if the asexuals eventually go extinct.

On the other hand, as discussed above, some asexuals may also have a positive effect on the distribution of their sexual hosts as they can transmit their genes far away from their own distribution. The examples are hybridogenetic frogs *P. esculentus* which serve as a “vector” transmitting clonal genomes of a parental species, *P. ridibundus*, to western Europe, far beyond its original range (Arano et al., 1994; Pagano et al., 2001), gynogenetic *Cobitis* hybrids that transmit genes of its Danubian parent, *C. elongatoides*, hundreds of kilometers outside its range to the Rhine River region and to southern Ukraine (Choleva et al., 2008), or *Corbicula* clams, whose asexual hermaphroditic reproduction increases invasive success (Pigneur et al., 2011, 2012). Similarly in plants, such as in the genus *Rubus*, polyploid apomicts may preserve ancestral alleles lost in their sexual ancestors during Pleistocene ice‐age bottlenecks and also spread younger alleles obtained from diploids via recent gene flow (Sochor et al., 2017).

## 5 CONCLUSIONS

Asexual organisms are worthy objects for studies by themselves, but their ecological and evolutionary influences on other species and biodiversity in general are perhaps even more important and should be more appreciated. Here, we listed a number of mechanisms of how the very existence of asexuals, in particular sperm‐dependent parthenogens, can have major effects on coexisting sexual species and biodiversity overall in terms of their (1) genetic architecture, (2) diversification and speciation, and (3) spatial distribution.

1. The genetic architecture of sexual species might be influenced in systems when a clonal genome finds its way into a sexual gene pool. Such asexual‐to‐sexual gene flow differs from gene exchange between sexual species because asexuals transmit their genetic material without recombination. The recipient sexual gene pool might be impacted negatively by introducing deleterious mutations but also positively by advantageous combinations of alleles that coevolved in linkage. Asexuals of hybrid origin can further serve as a bridge for introgression of alleles or whole mitochondrial genomes between sexual species. Even without asexual‐to‐sexual gene flow, extensive mating between sperm‐dependent parthenogens and their sexual hosts can reduce effective population size, increase the strength of genetic drift, and thus decrease genetic variability and efficiency of natural selection in sexual populations.
2. Sperm‐dependent parthenogens can contribute to differentiation of populations of sexual species by reducing their effective population size and increasing the strength of genetic drift but can also promote the accomplishment of speciation between hybridizing taxa. Since asexually reproducing hybrids restrict the gene flow between parental species to much higher extent than sexual hybrids, hybrid asexuality may be viewed as a special type of postzygotic incompatibility. It could be also predicted that sperm‐dependent parthenogens reinforce the formation of prezygotic barriers by exerting selection pressure on mate‐recognition systems in hybridizing sexual species, a process analogous to reinforcement in classical hybrid zones. Finally, asexuals can play an important role in speciation when, through an intermediate stage of polyploid forms, they can evolve into a new sexual species.
3. Asexuals may affect large‐scale biogeographic patterns of related sexual species. They frequently outcompete their sexual counterparts in disturbed habitats, at higher latitudes and altitudes, or at the edge of distribution ranges. Theoretical models also reveal that sperm‐dependent asexuals reduce abundance and density of sexual populations and thus may reduce expansion speed of sexual host species. In complexes composed of two sexual species and their sperm‐dependent hybrids, sperm‐dependent asexuals may significantly affect competition between sexual species by a process analogous to parasite‐mediated competition (or apparent competition) well known in classical host–parasite systems. Sperm‐dependent parthenogens, thus, might be important players in forming the structure of ecosystems.

Recent advances show that most proposed mechanisms do have support from empirical cases of asexual–sexual coexistence in nature. It indicates that even if the existence of individual clonal species may be ephemeral from an evolutionary point of view, their impact on sexual species likely lasts much longer than the existence of individual clonal lineages. These are all important population or species‐level effects that should be included in our research programs. We hope our review is going to stimulate further research into the questions we raise.

In our review, given our life‐long specialization, we admit the taxonomic bias toward animals, mainly vertebrates, but we strived to document examples from invertebrates and plants for most mechanisms we mentioned. A combined review of the plant and animal literature is indeed desirable but made exceedingly difficult by important differences in reproductive biology, as well as differences in terminology, that is used between zoological and botanical literature (e.g., Van Dijk, 2009). We also listed several empirical examples supporting the relevance of the discussed effects. We also point out that many such examples concerned our own work, but this was not out of vanity, but rather because the discussed aspects of asexual–sexual interactions have otherwise received little attention to date. Our review provides testable hypotheses with clear predictions that may be explored by other scientists in other systems, searching for potentially overlooked empirical examples.

We also hope that expanded research will create positive feedback for ecology and evolution in general as new tools are developed. As an example, consider testing the hypothesis that some current sexual organisms have passed through an asexual stage in their evolutionary history either because of an asexual bridge, or because they had an asexual phase in the course of speciation. This would require the development of novel analytical tools incorporating not only population genetic approaches but also explicit models of asexual hereditary patterns. Such tools may eventually not just help find influences of asexuals but will also improve tools for all of biology.

## GLOSSARY

**Allee effect:** a phenomenon describing a positive correlation between population size or density and the mean individual fitness of a population. A higher mean population fitness in more abundant or dense populations may be associated with better mate finding, better cooperation among individuals, greater ability to change the environment in favor of the species, or with a lower rate of inbreeding and higher genetic variability.

**Androgenesis:** a form of asexual reproduction in which the offspring carry nuclear chromosomes from only the male parent. When the female and male parents represent two different species, offspring have the nuclear genome of the paternal species but the cytoplasmic organelles (e.g., mitochondria) from the maternal species.

**Apparent competition:** a form of mostly negative indirect interactions between species that arise because they share a natural enemy (predators, parasites, pathogens, or herbivores). When such an indirect competition is driven by a shared parasite, it is called *parasite‐mediated competition*.

**Asexual reproduction:** reproductive mode in which an organism passes on its genome (or parts of it) clonally as a result of vegetative reproduction, polyembryony, or by circumventing recombination during gametogenesis. The latter is gametic asexuality.

**Clonal:** this makes reference to a mode of inheritance where all or most of the genome is passed on unaltered.

**Cybrids:** hybrids containing a nucleus of one species and cytoplasm of another species.

**Effective population size (*N***_**e**_ **):** reflects the rate at which genetic diversity will be lost following genetic drift, and this rate is inversely proportional to a population's *N*_e_. *N*_e_ is reduced by unequal sex ratio, variation in reproductive success, and by the fluctuation of the population size over time.

**Extended speciation continuum:** a conceptual framework linking the formation of asexual reproduction in hybrids with the classical speciation continuum assuming gradual formation of postzygotic reproductive barriers among diverging taxa. It posits that before accumulating genetic incompatibility between hybridizing species cause complete sterility or even inviability, they may occasionally distort hybrid's gametogenesis toward production of unreduced gametes, thereby sometimes alleviating problems in chromosomal pairing and rescuing hybrid's fertility, simultaneously triggering its (hemi‐) clonal reproduction.

**Gynogenesis:** a form of asexual reproduction in which females produce typically diploid eggs that are pseudo‐fertilized by sperm of males from a different species. The sperm genome is typically not incorporated, and inheritance is maternal.

**Hybridogenesis:** a form of asexual reproduction in which females produce haploid or diploid eggs that are usually fertilized by males from a different species. The sperm genome is incorporated and expressed but excluded from the germ line during gametogenesis. The female genome is, thus, inherited clonally.

**Kleptogenesis:** gynogenetic or hybridogenetic type of reproduction with occasional incorporation of the sperm‐derived genome into the otherwise clonal lineage. Sperm incorporation leads to genome addition (and ploidy elevation) or genome replacement (when the original maternal genome is discarded).

**Mixis:** well‐defined haploid and diploid phases in ontogenetic development that alternate.

**Muller's ratchet:** a process of the irreversible accumulation of deleterious mutations in a clonal genome because of the absence of recombination.

**Sexual reproduction:** prevailing mode of reproduction in eukaryotes, characterized by production of offspring via syngamy of meiotically produced gametes. Recombination and segregation of chromosomes (alleles) during meiosis result in genetically variable offspring.

**Sperm‐dependent parthenogenesis:** a form of clonal inheritance where eggs need to interact with sperm, either in gynogenesis or hybridogenesis. This is also called pseudogamy or sexual parasitism.

## AUTHOR CONTRIBUTIONS

**Karel Janko:** Conceptualization (lead); funding acquisition (lead); writing – original draft (equal). **Peter Mikulíček:** Conceptualization (equal); writing – original draft (equal); writing – review and editing (equal). **Roman Hobza:** Writing – original draft (supporting); writing – review and editing (supporting). **Ingo Schlupp:** Conceptualization (supporting); writing – original draft (supporting); writing – review and editing (equal).

## Data Availability

This is a review and contains no original data. No original data were used, this is a review paper. The data that support the findings of this study are available from the corresponding author upon reasonable request.
